# Review of Platonov’s “Sports Training Periodization. General Theory and its Practical Application” – Kiev: Olympic Literature, 2013

**DOI:** 10.2478/hukin-2014-0131

**Published:** 2014-12-30

**Authors:** Vladimir Lyakh, Kazimierz Mikołajec, Przemysław Bujas, Ryszard Litkowycz

**Affiliations:** 1 Department of Theory of Sport and Kinesiology, Academy of Physical Education in Cracow.; 2 Department of Team Sport Games, Academy of Physical Education in Katowice.

The reviewed book belongs to the most comprehensive and fundamental monographs concerning sports training periodization. The book was written by one of the most renowned modern specialists, a Ukrainian professor Platonov, whose books have been published all over the world, in 10 languages, in countries such as Argentina, Bulgaria, Brazil, Venezuela, Germany, Egypt, Spain, Italy, China, Poland, Russia, Tunisia, France and Japan. The author was awarded the Olympic Order by the International Olympic Committee in 2001 for his works on theory and methodology of sports training.

The author of the book attempts to systematize, generalize and analyse various scientific data concerning modern sports practices in the area of training periodization, and to present it as a comprehensive theory.

The book consists of six parts. The first part is divided into four chapters where the author analyses how the knowledge of training periodization developed throughout years and presents in detail the concept of periodization created by Matveyev, including the development of the concept shown in the works of authors from many countries of the world. Platonov emphasises that “Matveyev founded his concept on an objective generalization of some significant patterns in the development of peak performance, the study of sports training guidelines and a conceptual apparatus, not on “a description of mono, two and mixed-cycle variations of a year round preparation periodization”, as in his opinion, some specialists have been trying to do (Stone et al. 1981; Bompa 1992; Plick & Stone 2003; Issurin 2010; Haff & Haff 2012, and others). Therefore, the author points to the fact that “it is absolutely unfounded to artificially treat Matveyev as a supporter of exclusively monocycle periodization system of a year round preparation because such a system is inadequate for the needs of modern sports and does not guarantee athletes a successful performance in a greater number of competitions” (Issurin 2010; Bompa & Haff 2009; Haff & Haff 2012, and others). Platonov summarizes that Matveyev “never claimed that the right development of physical fitness can be based only on mono-, two- or three-cycle periodization of a year round preparation”. He only underlines that the duration of cycles and preparation periods should develop the abilities of an athlete to achieve a new, higher level of performance. In endurance sport disciplines, this task can be successfully undertaken after creating a plan based on a year round cycle; in strength-speed disciplines – a six-month-long plan based on shorter cycles is more appropriate. According to Matveyev, it is possible in some sport disciplines, but only under one condition: the preparatory phase (fundamental preparation) should be sufficiently long. During this time, an athlete should achieve a new level of fitness. If this period is shorter, this might turn out problematic. According to Matveyev, depending on specific sport practice conditions, many variations of the duration of monocycles and their periods are possible. But in all planning, a fundamental preparatory phase should be maintained and it should be long enough to enable an athlete to keep training effectively and achieve the desired result (Matveyev 1999, 2005). As Platonov emphasises, Matveyev’s views correspond with the preparation of most modern athletes, who are “not aiming at immediate success in second-league competitions, but at planned and effective preparation for the most important competitions, most of all Olympic Games and World Championships”. This opinion is fully supported by Platonov and a number of specialists in periodization of a year round preparation. The periodization theory was recognized not only by the majority of Soviet coaches, sports instructors and scientists working with high-class athletes, but it also attracted the attention of specialists from other countries in the world, mostly the so-called socialist countries of Eastern Europe and from Cuba. The period of 1970–1990, when this theory became the basis of year round preparation of national teams taking part in international competitions, it turned out to be most fortunate for athletes from the USSR, the German Democratic Republic and other socialist countries. Platonov underlines that the periodization theory developed by Matveyev was further expanded by specialists from Eastern Europe (Ozolin 1984; Nadory & Granek 1989; Zelazkow 1986; Harre 1971, 1982; Müller 1989, and others), which provided the possibility to create a system of a year round preparation directed both at improving general sports performance, as well as reaching peak performance for major competitions. Platonov’s observations point to the fact that in the USSR, members of many national teams achieved their best result of the season in major competitions in 55–70% of cases, what significantly exceeded the results of athletes from Western Europe.

At the same time, the scholars who backed Matveyev’s periodization theory and Matveyev himself (1991, 1998, 1999, 2005, 2010), whose last works are little known to the western scientists and coaches, underlined that training periodization is a dynamic and continuously developing area of knowledge. With the appearance of new data, constructive ideas and novel hypotheses, the periodization theory receives a new shape, and becomes better defined. The main contribution to creative development of the theory of periodization was made by Platonov himself (1997, 2004), which should not be overlooked, especially with the view of the fact that his main opponents (Werhoszanski 1985, 2005; Bondarczuk 2005; Issurin 2008, 2010, Bompa & Haff 1999, 2009, and others), who criticised Matveyev’s theory and presented another approach to this problem, in our opinion, forgot about Platonov’s views for a number of reasons.

In the 1990s, Platonov generalised his long-term scientific experience in exploration and practical implementation of periodization of year round preparation for high-class athletes and showed (1997, 2004) the possibilities to manage an extensive sports calendar with the use of periodization models based on 4–7 cycles. Platonov indicates that when creating such models, it seems fundamental to find a balance between the independence (individuality) of each macrocycle ending with a high priority competition and the macrocycle’s place in the whole system of a year round preparation aimed at achieving the highest results in a given year. Each macrocycle should be based on the training effects of the previous macrocycles and developed with the view that the performance in the main competition in a given macrocycle has to be relatively successful, but the training system should not break the guidelines of rational preparation for the main competition in a given year (Platonov 1997).

Building multicycle periodization models, which are meticulously analysed and discussed in the reviewed book, required from Platonov considerable changes of the traditional approach to preparation of different macrocycles in terms of their duration, general structure, load dynamics, interrelations between basic (fundamental) measures and special preparation, and also building a mesocycle of direct preparation for competitions. However, Platonov underlines that this correlation (extension and further definition) did not occur because the legitimacy and principles of the existing periodization theory were rejected, but because its empirical basis and theoretical part were expanded. He also emphasizes that even when creating optimal year round preparation programs based on multicycle periodization, it should be taken into consideration that successful performance in the end of every macrocycle might lead to a decrease of results in the main competition. However, the higher the number of macrocycles, the higher the likelihood of this result being lower (Platonov 2008). According to the author, if a multicycle year round preparation is created in this plan on the basis of “block periodization”, where every macrocycle is a “mini version of a year round cycle”, and an “independent stage” with a standard structure, as some specialists recommend (Issurin 2010; Haff 2012), that it is very possible that not only the main competition performance will be unsuccessful, but also the quality of preparation will be lower in various aspects.

In chapter 2 “Criticism of periodization theory, alternative and developing concepts and approaches”, Platonov does not only analyse the critical remarks on Matveyev’s periodization theory on a year round preparation of high-level athletes, but he also discusses alternative ideas and approaches proposed by Wierchoszanski (1985–2005), Worobjew (1989), Bondarczuk (2005), Issurin (2008, 2010) and western specialists (Stone et al. 1981, 1982, 2007; Fleck & Kreamer 1996, 2004; Sitt 2003; Baker 2007; Haff & Haff 2012; O’Bryant 2002; Plisk & Stone 2003; Stone 2004, Bompa 1994, 1999, 2002, 2006; Bompa & Haff 2009, and others). Unfortunately, it is impossible to present in one study the full list of fundamental objections concerning these authors’ ideas.

The problem of American approach to training periodization concerning preparation for the main competition was that “its study in most cases was based on a very limited data - only certain parts of preparation or selected motor abilities”. Platonov refers to the works of Plisk & Stone (2003), Brown, Greenwood (2005); Baker (2007); Stone et al. (2007), who make their conclusions on the basis of a detailed study of various strength training periodization in accordance with the demands of strength-speed oriented sport disciplines; in the works of Balague (2000) and Bompa (2002), the problem of mental preparation is analysed separately; the investigation of Bompa & Haff (2009) shows that the main purpose of periodization is increasing the efficiency of metabolic pathways.

Unfortunately, the works of American specialists concerning periodization “does not have sufficient empirical basis, they are not adequate to the complexity of the problem and the lack of theoretical studies has been recorded”. In order to illustrate that problem, Platonov refers to numerous examples of the most famous studies published in recent years (Plisk &, Stone 2003; Stone 2004; Stone et al. 2007).

Platonov analyses in detail the works of a Canadian scientist Bompa (Bompa 1994, 1999, 2002, 2006, Bompa & Carrera 2005; Bompa & Haff 2009). It could be expected from Bomba that he unified the approach to periodization, its conceptual and terminological base more than any other western specialist. It was predicted that his scientific activity made Western and Eastern views on this matter much closer to each other. However, Bomba chose his own path: he refused or rejected most of the theoretical knowledge and studies gathered in the countries of Eastern Europe. He revised the traditional and well-verified approach to the structure of preparation, fundamentally changed the terminology and the majority of specific sports training rules for numerous sports. Moreover, Bompa completely ignored the content of long-term scientific investigations, practical achievements of the best eastern European coaches in the last 25 years, as well as a major part of knowledge coming from western countries, including the USA. Consequently, in a lot of Bompa’s works, including the most recent ones (Bompa 2002, Bompa & Carrera 2005; Bompa & Haff 2009), some important theory concerning this issue is practically non-existent, even though until the modern times, this theory has been formulated in a sufficiently unified system of knowledge free of any contradictions. This had to create the situation that many of the mentioned practical recommendations have an abstract and contradictory character, especially when they are analysed regarding to the modern rules important for rational, effective periodization, knowledge concerning human biology and some current practical experience.

Prominent coaches from different countries have contributed significantly to the development of the American approach. They are actively involved in seeking out effective periodization models, which guarantee planned and effective preparation for the most prestigious competitions, especially Olympic Games and World Championships. Their views on this issue were formulated not only on the basis of materials concerning sports science, but also the experience in sports practice gathered in different countries, mostly in Eastern Europe. This approach can be observed in studies written by top-level coaches attempting to build their concepts on very serious scientific foundations (Maglischo 2003; Sweetenham & Atkinson 2003; Jochums 2005; Leonard 2008).

The third chapter of Platonov’s monography is dedicated to explaining the general structure, content and function of periodization theory and systemizing the conceptual-terminological basis in the area of sports training periodization.

In the fourth chapter, Platonov makes an attempt to systemize and validate special and general didactic rules of training periodization system. The most important specific rules of sports preparation, which, according to Platonov, have been verified in sport practice and scientifically developed, are as follows:

- orientation on major achievements;
- advanced specialization;
- compatibility of general (fundamental, basic) and special preparation;
- continuity of the training process;
- balance between accumulating and the tendency to maximise loads;
- variability of training loads;
- cyclicity of the preparation process;
- compatibility (uniformity) and interdependence of the structure of sports activities and preparation.

The second part of the monography entitled “General foundations of sports training periodization theory” consists of six chapters: chapter 5 “Adaptation in sport”, chapter 6 “Ontogenetic development of human being and adaptive processes”, chapter 7 “Skeletal muscles: structure, functions, adaptation”, chapter 8 “Systems of energy supply for muscle activity”, chapter 9 “Training Loads, fatigue, regeneration, supercompensation and long-term training effects”, and chapter 10 “Control of voluntary movements: basics”. Platonov himself emphasises that this part of the book contains extensive knowledge from related fields, mostly biology, and all types of theories and methodologically grounded scientific approaches.

In the third part “Megastructure of preparation process and system of permanent sport selection”, Platonov analyses the following issues: “Long-term preparation: developing the system of knowledge” (chapter 11), “Regularities and specific characteristics of long-term training” (chapter 12), “Modern concepts of long-term periodization” (chapter 13), “Main selection guidelines in the system of long-term preparation” (chapter 14). Megastructure is understood as the structure of a long-term multiannual preparation and its stages: four-year Olympic cycles. Here Platonov clearly distinguishes two independent periods: the period of achieving championship level (it usually lasts from 7–8 to 10–12 years) and the period of maintaining top performance (from 2–3 to 10–15 years or more). He divides the first stage into four independent phases: 1) initial preparation, 2) basic preparation, 3) specific preparation, 4) preparation for top performance. According to Platonov, the second stage comprises of three substages: maximal development of individual motor abilities and skills, maintaining peak performance and the process of gradual decrease of achievements.

The fourth part of the monography entitled “Micro - and mesostructure of the training process” consists of four chapters: chapter 15 “Exercises for sports training”, chapter 16 “Warm-up and its structure”, chapter 17 “The structure of training session”, chapter 18 “Planning the Microcycles”, chapter 19 “Planning the mesocycles”.

In this part, Platonov presents the structure of different microcycles, practice sessions, as well as series of exercises included in the training program. He distinguishes and describes the following 5 types of microcycles: introductory, striking, transforming, regenerative and competitive. Training sessions are divided into several types according to their aim: theoretical, practical, theoretical-practical, regenerative, modelling and controlling. They are extensively described in the book. Platonov also presents the description of basic training models, such us: individual, group, team and circuit training. Depending on the training load three levels were selected: high, medium and low. Considering the training importance it can be divided into main and supplementary. Finally, depending on its purpose the author presents two types: oriented on selected specific skills or comprehensive.

Platonov shows the mesostructure (Greek: mesos - medium) as a structure of mesocycles. He describes and analyses the following mesocycles: introductory, basic, regenerative-preparatory, regenerative-maintaining. Each of them is specifically oriented. Using numerous examples, Platonov demonstrates the best connection of microcycles in mesocycles of different types with the aim of preparing the participants to achieve best sport results. One of them is shown in table 1. It indicates that connecting different types of microcycles in a mesocycle does not only depend on the type of a mesocycle, but also on tasks established for a given period, or the stage of multiannuall or year round preparation, as well as on an athlete’s specialization, his/her qualifications and their level of preparation.

Platonov also draws our attention to the specific character of mesocycles in training female athletes. Referring to the study of Fox et al. (1993), Szachlinoj (2001, 2002) and Lisicka (1982), he demonstrated that it is advantageous to take into consideration specific characteristics of women, for example their menstrual cycle, which allows to make the training program more effective.

The review of the second part of Platnonov’s monography will be continued in the next issue.

## Figures and Tables

**Table 1:** Cumulative load of microcycles in various types of mesocycles (high-level athletes)

Type of mesocycle	Type of microcycle and cumulative training loads			
	I	II	III	IV
Introductory	Introductory – average training load (high intensity practice sessions are not planned)	Introductory – average training loads (1 practice session with high intensity)	Striking– high intensity (3 practice sessions with high intensity)	Regenerative – low training loads
Basic	Striking– high intensity (4 practice sessions with high intensity)	Striking– high intensity (3 practice sessions with high intensity)	Striking– high intensity (5 training sessions with high intensity)	Regenerative – low training loads
Control-preparatory	Striking– high intensity (5 practice sessions with high intensity)	Regenerative – low training loads	Striking– high intensity (5 training sessions with high intensity)	Regenerative – low training loads
Precompetitive	Regenerative – high load practice sessions are not planned	Striking– high intensity (2 practice sessions with high intensity)	Transforming – average loads (1 training session with high intensity	Regenerative – low training loads
Competitive	Transforming – average loads (1 practice session with high intensity)	Competitive –low training loads, Competitive loads depands on competition schedule	Transforming – low training loads	Competitive –low training loads, Competitive loads depands on competition schedule

